# Transmission Routes for Nipah Virus from Malaysia and Bangladesh

**DOI:** 10.3201/eid1812.120875

**Published:** 2012-12

**Authors:** Bronwyn A. Clayton, Deborah Middleton, Jemma Bergfeld, Jessica Haining, Rachel Arkinstall, Linfa Wang, Glenn A. Marsh

**Affiliations:** Author affiliations: Commonwealth Scientific and Industrial Research Organisation Livestock Industries, Geelong, Victoria, Australia (B.A. Clayton, D. Middleton, J. Bergfeld, J. Haining, R. Arkinstall, L. Wang, G.A. Marsh);; University of Melbourne, Parkville, Victoria, Australia (B.A. Clayton);; Duke–National University of Singapore Graduate Medical School, Singapore (L. Wang)

**Keywords:** Nipah virus, ferret, pathogenesis, shedding, Bangladesh, Malaysia, transmission, viruses

## Abstract

Human infections with Nipah virus in Malaysia and Bangladesh are associated with markedly different patterns of transmission and pathogenicity. To compare the 2 strains, we conducted an in vivo study in which 2 groups of ferrets were oronasally exposed to either the Malaysia or Bangladesh strain of Nipah virus. Viral shedding and tissue tropism were compared between the 2 groups. Over the course of infection, significantly higher levels of viral RNA were recovered from oral secretions of ferrets infected with the Bangladesh strain. Higher levels of oral shedding of the Bangladesh strain of Nipah virus might be a key factor in onward transmission in outbreaks among humans.

## Abstract 2

Nipah virus infection in humans is associated with a higher death rate in Bangladesh than in Malaysia. Additionally, Nipah virus spreads from person to person in Bangladesh but not in Malaysia. To investigate why these differences occur, researchers looked for differences in the virus strains from each country. In experimentally infected ferrets, they examined which tissues each strain infected and how each strain was excreted from the body. They found higher concentrations of the Bangladesh strain in secretions from the mouth. Increased oral excretion of the Bangladesh strain in humans might explain why person-to-person transmission of Nipah virus occurs in that region.

Nipah virus (NiV), a bat-borne paramyxovirus, has caused outbreaks of human disease with high mortality rates in Malaysia, Singapore, India, and Bangladesh. Two divergent NiV strains (NiV-Malaysia and NiV-Bangladesh) share 91.8% nt sequence identity (*1*).

NiV-Malaysia emerged in 1998 during an outbreak of infectious respiratory and neurologic disease in commercially farmed pigs, presumably after virus spillover from Malaysian flying foxes (*2*). Pigs were the source of infection for farm and abattoir workers, resulting in a widespread outbreak of severe febrile encephalitic disease among humans (*3*–*5*); >250 cases were reported in Malaysia and Singapore, and the case-fatality rate approached 40% (*2*,*5*,*6*). No cases of human-to-human transmission were reported during the outbreak (*7*,*8*). However, rare instances of human-to-human transmission have been suggested by asymptomatic seroconversion against NiV-Malaysia in a health care worker, which was recognized after the outbreak (*9*), and by a recently reported case of late-onset NiV encephalitis attributed to transmission from infected family members (*10*).

NiV-Bangladesh emerged in 2001 in Bangladesh (*11*,*12*), and subsequent outbreaks of disease have occurred almost annually (*12*–*20*). Since 2001, >200 cases in humans have been identified in Bangladesh; the overall case-fatality rate is >70% (*21*). In contrast to the rare instances of human-to-human transmission of NiV-Malaysia, human-to-human transmission of NiV-Bangladesh is a major pathway for human infection (*13*).

The different transmission characteristics of NiV-Malaysia and NiV-Bangladesh might be attributable to differences in infectivity and pathogenicity of virus strains and in tissue tropism, reflected by higher incidence of respiratory disease in NiV-Bangladesh–infected patients (*14*,*21*). We assessed the role that tissue tropism and shedding characteristics of NiV-Malaysia and NiV-Bangladesh might play in clinical outcomes and increasing transmission risk. For this purpose, we used a mammalian infection model, the ferret, in which NiV causes fulminating systemic disease, with fever and neurologic and/or respiratory signs, similar to those in humans (*15*). Here we describe a ferret model for NiV-Bangladesh infection and our comparison of the characteristics of infections caused by NiV-Malaysia and NiV-Bangladesh in the ferret.

## Materials and Methods

### Animal Infection, Handling, and Housing

A total of 15 male ferrets, 12–18 months of age, were oronasally exposed to 5,000 50% tissue culture infective doses of low-passage isolates of Nipah virus from humans. Animals were randomly assigned to receive NiV-Bangladesh (n = 8, ferrets B1–B8) or NiV-Malaysia (n = 7, ferrets M9–M15). The 2 groups were housed under separate biosafety level 4 conditions as described (*17*). The specific NiV-Bangladesh isolate was Nipah Bangladesh/human/2004/Rajbari, R1, which came from the oropharynx of 1 of 12 patients infected during an outbreak of NiV encephalitis in Rajbari district, Bangladesh (*1*,*16*). The patient was a 10-year-old boy with neurologic disease and respiratory involvement characterized by coughing, wheezing, and difficulty breathing (S. Luby, pers. comm.). The specific NiV-Malaysia isolate was Nipah virus/Malaysia/human/99, which came from the cerebrospinal fluid of a patient with encephalitis. We selected a challenge dose that was expected to infect all exposed animals with NiV-Malaysia (*15*) and NiV-Bangladesh (D. Middleton, unpub. data). Ferrets were anesthetized as described before viral challenge and for subsequent sample collection (*17*). Procedures involving live animals were approved by the Commonwealth Scientific and Industrial Research Organisation, Australian Animal Health Laboratory, Animal Ethics Committee.

### Animal Monitoring and Sampling

After receiving the challenge dose, animals were assessed daily for clinical signs of disease. Every 48 hours, nasal wash samples, oral and rectal swab samples, and blood (axillary vein) were collected and temperature and weight were recorded. Sampling days were staggered so that sampling of ferrets B1–B4 and M9–M11 started on 1 day postinfection (dpi) and sampling of ferrets B5–B8 and M12–M15 started on 2 dpi. Environmental samples of urine and feces were obtained daily from cage floors.

Ferrets were euthanized at predetermined humane end points as described (*15*). Clinical samples and various tissues were collected immediately before euthanasia or during postmortem examination.

### Sample Collection, Processing, and Analysis

Nasal wash, swab, urine, whole blood (EDTA treated), tissue, and fecal samples were collected and processed in the same manner as tissue samples and then used for virus isolation, RNA extraction, and TaqMan reverse transcription PCR (RT-PCR; selective for the NiV N gene) as described (*17*–*19*). Samples with a mean NiV N gene cycle threshold value <39.1 were defined as positive for NiV RNA. For tissue samples, NiV N gene values were normalized to host cell 18S rRNA by multiplex RT-PCR as described (*22*) but by using probe (5′-VIC-TGCTGGCACCAGACTTGCCCTC-TAMRA-3′). Tissues were also processed for histopathologic and immunohistochemical examination with rabbit α-NiV N protein antiserum (*20*).

### Statistical Analysis

To compare trends in virus shedding over time, we analyzed transformed RT-PCR data from nasal, oral, and rectal swab samples by using a residual maximum-likelihood (REML) model in GenStat statistical software, version 3 (VSN International, Hemel Hempstead, UK). Data were collapsed into 48-hour periods, thereby generating 4 time points for comparison: dpi 1–2, 3–4, 5–6, and 7–8. We omitted dpi 9–10 from analysis because few animals in either group survived this long. To analyze the trend of shedding over time for each virus, we fitted the interaction of virus and days to the model. 

On the basis of REML analysis outcomes, we also estimated the amount of NiV-Bangladesh and NiV-Malaysia shed by individual animals over the course of infection by calculating the area under the curve (AUC) for viral RNA (by using the trapezoidal rule) for nasal wash and oral and rectal swab samples. Blood samples were similarly assessed. Estimates were transformed to the log_10_ scale, and mean AUCs for NiV-Bangladesh (n = 8) and NiV-Malaysia (n = 7) samples were compared by using an independent-samples *t* test.

At the time of euthanasia, we compared levels of viral RNA in nasal, oral, rectal, urine, and blood samples between the 2 groups by using independent-sample *t* tests of transformed data. Also at the time of euthanasia, we similarly assessed levels of viral RNA in tissue. Analysis by *t* test did not assume equal differences. All tests used were 2-sided, and p<0.05 was defined as statistically significant.

## Results

### Clinical Features of Infection

Clinical signs were those of lower respiratory tract and neurologic system infection. Clinical signs were similar for all 8 ferrets challenged with NiV-Bangladesh and for 6 of 7 challenged with NiV-Malaysia (Table 1). In 1 ferret challenged with NiV-Malaysia (ferret M11), localized bacterial lymphadenitis (confirmed by histopathologic examination) developed, and the ferret was euthanized on humane grounds at 5 dpi, at which time no clinical signs of NiV infection had been observed. The first sign of disease was pyrexia (rectal temperature ≥40°) for most animals; disease progressed rapidly to its humane end point within 72 hours of pyrexia onset.

**Table 1:** Clinical disease in ferrets after experimental infection with NiV from Bangladesh or Malaysia*

NiV type and ferret no.	Euthanasia, dpi	Resp†	Neuro	Hemorr‡	Criteria for euthanasia	Clinical signs
Bangladesh						
B1	7	**–**	**–**	**–**	Obtundation	Severely obtunded; hunched posture
B2	7	**+**	***+/−***	**–**	Respiratory +/− mild neurologic signs	Hunched posture; possible mild neurologic disease (agitation); sneezing; >10% reduction in body weight§
B3	7	**+**	**+/−**	**–**	Respiratory +/− neurologic signs	Possible mild neurologic disease (continuous licking, smacking lips); dehydration;¶ vomiting; rapid deterioration in clinical condition after sampling at 7 dpi
B4	7	**+**	**+**	**–**	Respiratory signs, neurologic signs, and obtundation	Fine tremors/myoclonus of forelimbs; nasal discharge
B5	8	**–**	**+**	**–**	Neurologic signs and obtundation	Hind limb myoclonus/paresis, ataxia; dehydration; periorbital/facial/ventral neck edema
B6	9	**+**	**+**	**+**	Respiratory signs, neurologic signs, hemorrhage, and obtundation	Forelimb myoclonus; sneezing; mucoid nasal discharge; reduced feces production; periorbital/facial edema; hemorrhage of oral mucosa at euthanasia time point; >10% reduction in body weight
B7	8	**–**	**+**	**–**	Neurologic signs	Myoclonus and muscular spasm affecting the tail, ataxia; ventral neck edema
B8	8	**+**	**+**	**–**	Respiratory signs, neurologic signs, and obtundation	Myoclonus of the flanks, ataxia, hind limb paralysis; vomiting; ventral neck edema
Malaysia						
M9	7	**+**	**+**	**+**	Respiratory signs, neurologic signs, hemorrhage, and obtundation	Severe ataxia, facial and hind limb tremors, head tilt and torticollis (left); sneezing; nasal discharge; facial edema; hemorrhage of rectal mucosa at euthanasia
M10	7	**+**	**+**	**+**	Respiratory signs, neurologic signs, hemorrhage, and obtundation	Dyspnea with prolonged expiration phase; mild ataxia; reduced feces production; facial and ventral neck edema; hemorrhage from nose and mouth at euthanasia
M11	5	NA	NA	NA	NA	Euthanasia at 5 dpi for humane reasons; no evidence of clinical disease associated with NiV infection
M12	8	**–**	**+**	**+**	Neurologic signs and severe hemorrhage	Spastic paralysis of right forelimb, rhythmic myoclonus of right trunk, ataxia; sneezing; nasal discharge; facial edema; extensive cutaneous petechial hemorrhages and facial bruising
M13	9	**+**	**+/−**	**+**	Respiratory signs, hemorrhage, and obtundation	Mild neurologic disease (hind limb paresis) at 6 dpi but not apparent at euthanasia; nasal discharge; facial edema; inappetence; >10% reduction in body weight
M14	8	**–**	**+**	**+**	Neurologic signs, hemorrhage, and obtundation	Hunched posture; spastic paralysis of hind limbs, fine muscular fasciculations over flanks, ataxia; hunched posture; dehydration; nasal discharge; cutaneous petechial hemorrhages; >10% reduction in body weight
M15	10	**–**	**+**	**+**	Neurologic signs, hemorrhage, and obtundation	Sporadic hind limb myoclonus; recumbency; nasal discharge; cutaneous petechial hemorrhages and hemorrhage from mouth; >10% reduction in body weight

*NiV, Nipah virus; dpi, days postinfection; resp, respiratory involvement; neuro, neurologic involvement; hemorr, hemorrhage; +/–, vague clinical signs that might indicate neurologic involvement; NA, not applicable. †Increased respiratory effort and/or rate unless otherwise stated under clinical disease. ‡Cutaneous hemorrhages or frank hemorrhage from oral, nasal, and rectal mucosa. §Based on weight data collected before experimental infection. ¶Based on the observation of reduced skin turgor at physical examination.

Hemorrhage was another clinical sign, but it differed between the 2 groups. At the terminal stage of disease, NiV-Malaysia–infected animals experienced cutaneous petechial hemorrhage, accompanied by bleeding from oral, nasal, and rectal mucosa; whereas, only 1 NiV-Bangladesh–infected animal experienced bleeding (of the oral mucosa at the time of euthanasia).

### Virus Loads

**Figure 1:**
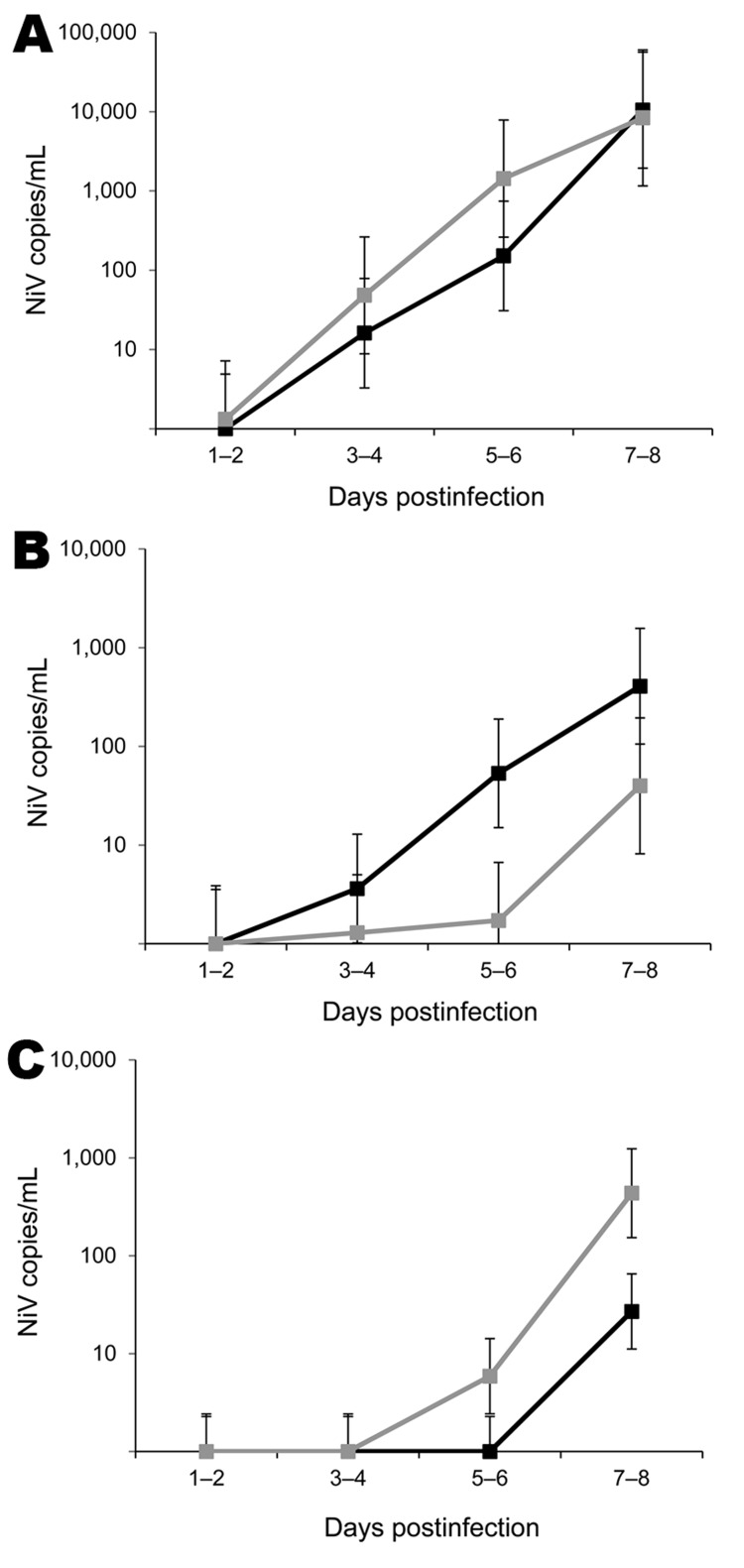
Predicted means for detection of Nipah virus (NiV) RNA in nasal wash samples (A), oral swab samples (B,) and rectal swab samples (C) from experimentally infected ferrets over time, based on residual maximum-likelihood analysis. Black line, NiV-Bangladesh; gray line, NiV-Malaysia. NiV N gene copies per milliliter of sample were calculated from reverse transcription data, then the transformation log_10_(x_1_ + 780) was calculated, where x_1_ = NiV gene copies per milliliter. Values were fitted in the residual maximum-likelihood model by using transformed data and are plotted as values relative to the original scale (y-axis; logarithmic scale). Error bars represent approximate upper and lower limits for 95% CIs for the mean (calculated as mean ± 2 SE relative to the transformed scale).

Viral RNA and virus isolation results are presented in Tables 2 and 3. Viral RNA was recovered from clinical samples from all animals with clinical disease, and virus was isolated from some samples. For ferret M11, viral RNA was detected in clinical samples as early as 3 dpi and in blood, indicating a productive infection after experimental challenge; data for ferret M11 were therefore included in analysis of shedding. RNA was detected in nasal, oral, or rectal samples from similar numbers of animals exposed to NiV-Malaysia or NiV-Bangladesh (by linear mixed-model analysis; data not shown). Mean levels of viral RNA in clinical samples increased throughout the course of infection and were highest at 7–8 dpi, the time of onset of severe clinical disease for most animals (Figure 1). In 4 NiV-Bangladesh–infected animals and all NiV-Malaysia–infected animals, viral RNA was detected in nasal secretions at least 24 hours before it was detected in blood and/or before onset of pyrexia.

**Table 2:** Viral RNA and virus isolation results from ferrets experimentally infected with Nipah virus, Bangladesh strain*

Ferret no., sample	Virus in shedding samples and blood over time, RNA/virus isolation†									
	Dpi 1	Dpi 2	Dpi 3	Dpi 4	Dpi 5	Dpi 6	Dpi 7	Dpi 8	Dpi 9	Dpi 10
B1										
NW	–		Indet		+/−‡		+/+			
OS	–		–		–‡		+/−			
RS	–		Indet		–‡		+/−			
Blood	–		+/‡		–‡		+/§			
B2										
NW	–		+/−		+/−		+/+			
OS	–		–		+/−		+/−			
RS	–		–		–		+/−			
Blood	–		–		+/+		+/§			
B3										
NW	–		+/−		–‡		+/−			
OS	–		–		–‡		+/−			
RS	–		–		–‡		+/+			
Blood	–		Indet		–‡		+/§			
B4										
NW	–		+/−		+/−		+/+‡			
OS	–		–		+/−		+/−‡			
RS	–		–		–		–‡			
Blood	–		–		–		+/§			
B5										
NW		–		+/−		+/+		+/+		
OS		–		+/+		+/−		+/+		
RS		Indet		–		–		+/−		
Blood		Indet		–		+/+		+/+		
B6										
NW		–		–		+/−‡		NS	+/+	
OS		–		–		+/+‡		NS	+/−	
RS		–		–		–‡		NS	+/−	
Blood		–		–		+/+‡		NS	+/−	
B7										
NW		–		+/−		+/+		+/+‡		
OS		–		+/+		+/+		+/−‡		
RS		–		–		–		+/+‡		
Blood		+/+		–		+/−		+/+‡		
B8										
NW		–		–		–‡		+/+		
OS		–		–		+/−‡		+/−		
RS		–		–		–‡		+/+		
Blood		–		–		+/+‡		+/+		

*Dpi, days postinfection; NW, nasal wash; –, Nipah virus not detected by reverse transcription PCR (RT-PCR) for N gene; therefore, virus isolation was not attempted for this sample; empty cells represent days on which sampling was not scheduled for that animal; Indet, mean cycle threshold value >39.1 (the defined cutoff value for positive samples); +/−, virus detected by RT-PCR but negative by virus isolation; +/+, sample positive by RT-PCR and virus isolation; OS, oral sample; RS, rectal sample; NS, not sampled. †RNA detection by RT-PCR. ‡Pyrexia first detected. §Virus isolation not attempted for this sample.

**Table 3:** Viral RNA and virus isolation results from ferrets experimentally infected with Nipah virus, Malaysia strain*

Ferret no., sample	Virus in shedding samples and blood over time, RNA/virus isolation†

Dpi 1	Dpi 2	Dpi 3	Dpi 4	Dpi 5	Dpi 6	Dpi 7	Dpi 8	Dpi 9	Dpi 10
M9										
NW	–		+/−		+/−‡		+/+			
OS	–		+/+		–‡		+/−			
RS	–		–		+/+‡		+/−			
Blood	–		–		+/−‡		+/+			
M10										
NW	–		+/−		+/+‡		+/+			
OS	–		–		–‡		+/+			
RS	–		–		+/−‡		+/−			
Blood	–		–		–‡		+/+			
M11										
NW	–		+/−		+/+					
OS	–		–		+/−					
RS	–		–		–					
Blood	–		–		+/+					
M12										
NW		+/+		+/+‡		+/−		+/+		
OS		–		–‡		Indet		+/−		
RS		–		–‡		+/−		+/+		
Blood		–		–‡		+/+		+/+		
M13										
NW		–		+/−		+/+‡		NS	+/+	
OS		–		–		–‡		NS	+/+	
RS		–		–		+/+‡		NS	+/−	
Blood		–		–		–‡		NS	+/+	
M14										
NW		–		+/−		+/+‡		+/+		
OS		–		–		+/−‡		+/+		
RS		–		–		–‡		+/−		
Blood		–		–		+/+‡		+/+		
M15										
NW		–		+/−		+/+		+/−‡		+/+
OS		–		–		+/−		–‡		+/+
RS		–		–		+/−		+/§		+/−
Blood		–		–		+/+		+/+‡		+/+

*Dpi, days postinfection; NW, nasal wash; –, Nipah virus not detected by reverse transcription PCR (RT-PCR) for N gene; therefore, virus isolation was not attempted for this sample; empty cells represent days on which sampling was not scheduled for that animal; +/−, virus detected by RT-PCR but negative by virus isolation; +/+, sample positive by RT-PCR and virus isolation; OS, oral sample; RS, rectal sample; Indet, mean cycle threshold value >39.1 (the defined cutoff value for positive samples); NS ,not sampled †RNA detection by RT-PCR. ‡Pyrexia first detected. §Virus isolation not attempted for this sample.

**Figure 2:**
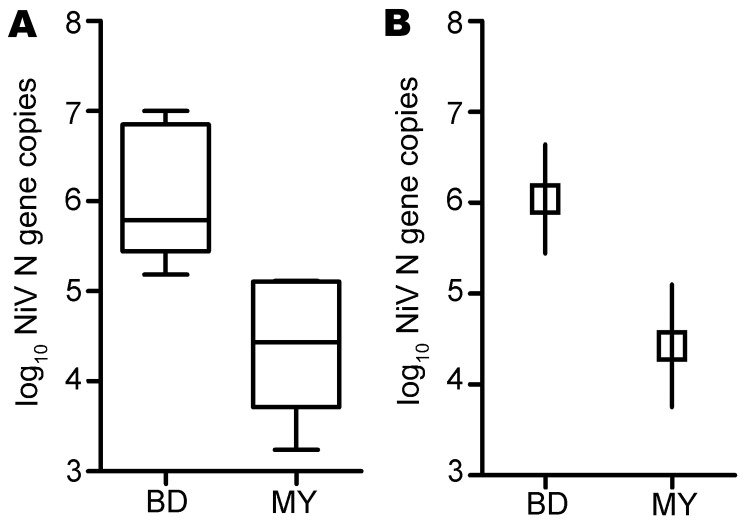
Oral shedding of Nipah virus (NiV) in experimentally infected ferrets. A) Results of viral RNA area under the curve (AUC) calculation. Lower margin, inner line, and upper margin of the boxes represent 25th percentiles, medians, and 75th percentiles, respectively. Whiskers show maximum and minimum values for each group. B) Comparison of mean AUC between NiV-Bangladesh and NiV-Malaysia. Mean AUC for the NiV-Bangladesh infection group was significantly higher than that for the NiV-Malaysia–infected group; p = 0.001. Interval bars represent 95% CIs for the means. BD, Bangladesh; MY, Malaysia.

 Levels of viral RNA were significantly higher in oral secretions from NiV-Bangladesh–infected than from NiV-Malaysia–infected animals; predicted mean viral RNA levels were at least 10-fold higher in the NiV-Bangladesh–infected group at 5–6 and 7–8 dpi (Figure 1; REML analysis; p = 0.038 for virus by days), and mean AUC was >30-fold higher (Figure 2; p = 0.001; t_12_ = 4.3). However, the rate of virus isolation from oral swab samples did not differ significantly between infection groups (Z test of 2 proportions; data not shown). NiV-Bangladesh was isolated from oral swab samples from ferrets B5 (4 dpi and at euthanasia), B6 (6 dpi), and B7 (4 and 6 dpi). Oral swabs from ferrets infected with NiV-Malaysia yielded isolates in ferret M9 at 3 dpi and in ferrets M10, M13, M14, and M15 at euthanasia (Table 3).

Nasal shedding of viral RNA for both viruses over time was higher than oral and rectal shedding (Figure 1). However, the shedding trend over time was similar between the 2 groups, and no significant difference was found between the mean AUCs (data not shown).

Rectal shedding of viral RNA was observed for most animals with clinical disease in both groups. Predicted means for rectal shedding were higher for the NiV-Malaysia–infected group at 5–6 and 7–8 dpi; for the NiV-Bangladesh–infected group, detection of viral RNA in rectal swab samples was delayed until 7–8 dpi (Figure 1; REML analysis p = 0.006 for virus by days). However, the total amount of viral RNA shed in rectal swab samples over the course of infection did not differ significantly between the 2 viruses (by AUC analysis; data not shown).

Viral RNA was generally detected in blood from 5 dpi on (Tables 2, 3). AUC analysis did not demonstrate a difference between the groups in total viral RNA in blood over the course of infection (data not shown).

Urine collection was rarely achieved by manual bladder expression during the course of infection. However, at the time of euthanasia, small volumes of urine were collected from all animals in the NiV-Bangladesh–infected group and from 5 of 6 animals in the NiV-Malaysia–infected group. The rate of detection of viral RNA and isolation of virus in urine was similar for each group (data not shown).

Environmental urine and fecal samples from both groups were NiV positive by RT-PCR and by virus isolation over the course of infection from as early as 3 dpi for 1 cage in the NiV-Malaysia–infected group (Table 4). The rate of detection and isolation of NiV from environmental samples was highest at 7 dpi, coinciding with onset of severe clinical disease in most animals.

**Table 4:** NiV in environmental samples after experimental infection of 15 ferrets*

Virus strain, cage no. (ferret no.)	log_10_ NiV copies/mL†																												
	Dpi 1			Dpi 2			Dpi 3			Dpi 4			Dpi 5			Dpi 6			Dpi 7			Dpi 8			Dpi 9			Dpi 10	
	U	F		U	F		U	F		U	F		U	F		U	F		U	F		U	F		U	F		U	F
Bangladesh																													
1 (B1, B2)	–	–		–	–		–	–		–	–		–	–		–	–		5.1‡	–		NA	NA		NA	NA		NA	NA
2 (B3, B4)	–	–		–	–		–	–		–	–		NS	NS		–	–		5.2‡	4.1		NA	NA		NA	NA		NA	NA
3 (B5, B6)	–	–		–	–		–	–		–	–		–	–		–	–		4.1	3.4		4.6‡	–		5.3‡	4.6		NA	NA
4 (B7, B8)	–	–		–	–		–	–		–	–		–	–		–	–		3.6	4.3		–	4.9‡		NS	–		NA	NA
Malaysia	–	–		–	–		–	–		–	–		–	–		–	–		–	–		–	–		–	–		NA	NA
5 (M9)	–	–		–	–		–	–		–	–		–	–		–	–		4.9‡	–		NA	NA		NA	NA		NA	NA
6 (M10, 11)	–	–		–	–		–	3.6‡		–	–		–	–		–	–		4.9‡	–		NA	NA		NA	NA		NA	NA
7 (M12, 13)	–	–		–	–		–	–		–	–		–	–		5.1‡	–		–	–		4.9‡	–		–	–		NA	NA
8 (M14, 15)	–	–		–	–		–	–		–	–		–	–		–	–		–	–		4.6‡	–		3.6	–		–	–

*NiV, Nipah virus; dpi, days postinfection; U, urine; F, feces; –,negative; NA, not applicable because cage was empty after euthanasia of ferrets; NS, no sample available. †Calculated from standard curve generated for NiV N gene copies by reverse transcription PCR. Samples with mean cycle threshold ≤39.1 (based on duplicate reactions) were defined as NiV positive. ‡Sample was also NiV positive by virus isolation.

### Histopathologic and Immunohistochemical Findings

#### NiV-Bangladesh

**Figure 3:**
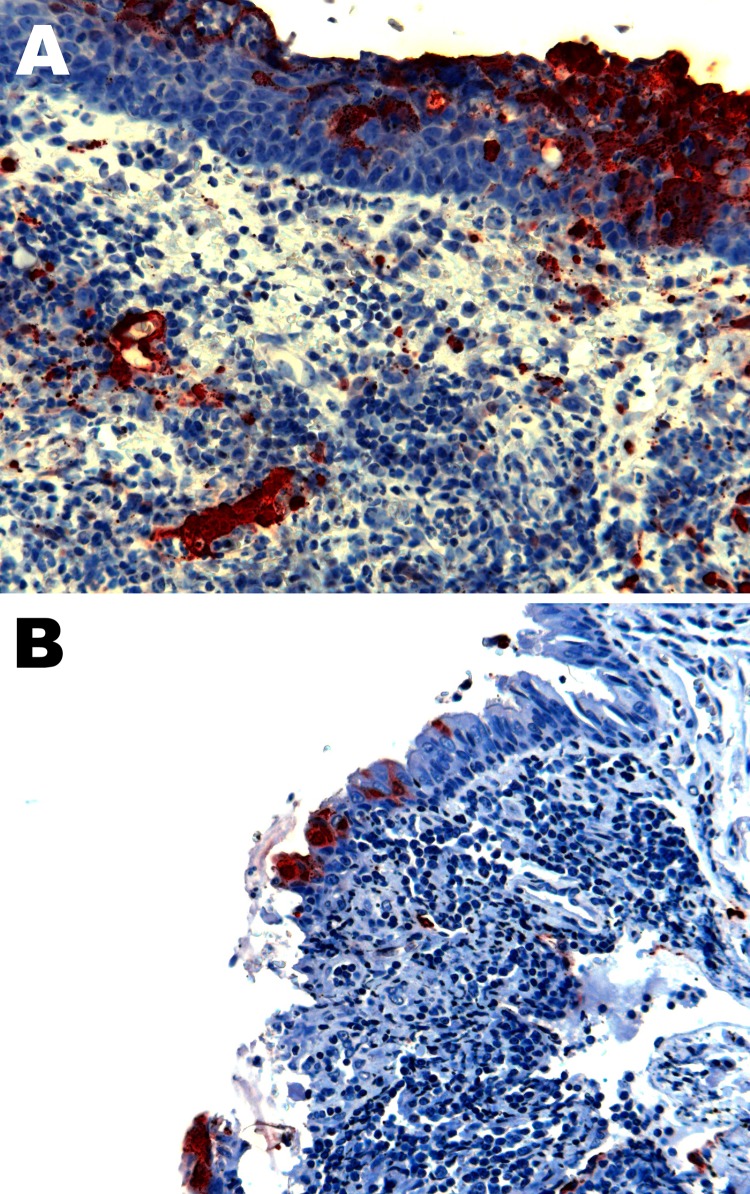
Nipah virus (NiV) antigen in acutely inflamed tonsillar tissue and overlying epithelium (A) and nasopharyngeal epithelium (B) in 2 ferrets infected with NiV-Bangladesh. Rabbit α-NiV N protein antiserum. Original magnification ×200.

**Figure 4:**
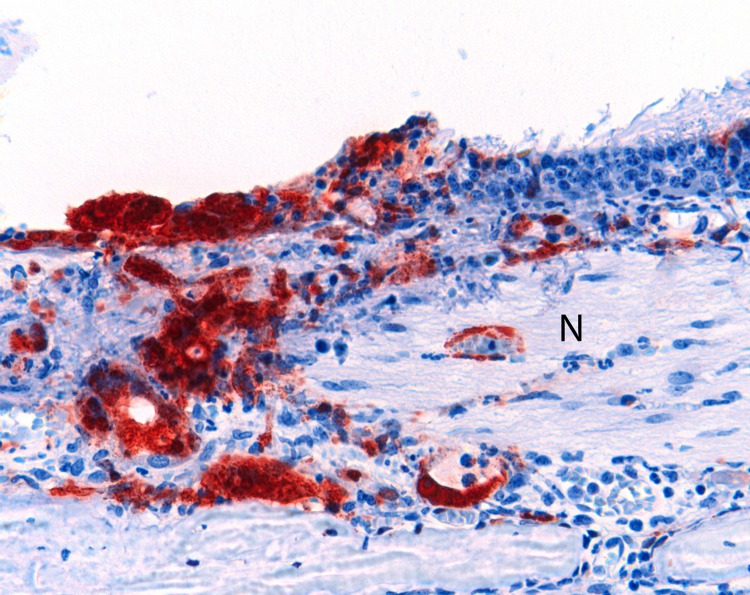
Olfactory epithelium of a ferret infected with Nipah virus (NiV)-Bangladesh. NiV antigen was observed in close association with submucosal nerve fibers (N). Rabbit α-NiV N protein antiserum. Original magnification ×200.

The main histopathologic findings for ferrets from both groups are summarized in Table 5. In ferrets exposed to NiV-Bangladesh, multisystemic inflammatory lesions developed, most consistently affecting the upper and lower respiratory tract, lymphoid tissue, kidneys, and liver. Lesions comprised mild to severe acute necrotizing rhinitis affecting olfactory and respiratory epithelium, focal necrotizing bronchoalveolitis, and marked lymphadenitis (most notably involving the submandibular and retropharyngeal lymph nodes, caudal cervical lymph nodes, and associated peritracheal and periesophageal lymph vessels). Tonsillitis and nasopharyngitis were also noted. In some animals, lymph node lesions were confined to subcapsular and cortical regions; in others, the entire nodal architecture was effaced. There was also glomerular necrosis with hyaline tubular casts; focal proximal renal tubular necrosis and interstitial nephritis; focal adrenal, splenic, and hepatic necrosis; and mild esophagitis and tracheitis. Vasculitis was detected in nasal submucosae, lungs, lymph nodes, spleen, and testes; syncytial cells of epithelial (bronchiole, renal tubule), endothelial (lymph node, testis), and unknown derivation (spleen, lymph node) were also identified. Viral antigen was found in tissues from each animal, including tonsillar (Figure 3, panel A) and nasopharyngeal (Figure 3, panel B) epithelium; vascular endothelium; syncytia; foci of inflammation in lung, bronchial, and bronchiolar epithelium; necrotic areas within lymphoid tissues and adrenal glands; necrotic glomeruli and renal tubular cells; necrotic hepatic acinar tissue; and the esophageal exudate from 1 animal. Within the nasal cavity, viral antigen was identified not only in respiratory and olfactory epithelium but also adjacent to submucosal nerve fibers in 2 animals (Figure 4). Mild testicular degeneration was observed in all animals and was attributed to fever.

**Table 5:** Histopathologic and immunohistochemical findings in major systems of ferrets infected with NiV from Bangladesh or Malaysia*

System, predominant lesion	No. animals with lesion/antigen/vasculitis†	
	NiV-Bangladesh, n = 8	NiV-Malaysia, n = 7
Respiratory		
Acute rhinitis	7/5/1	6/7/0
Acute bronchoalveolitis	8/8/1	7/7/4
Lymphoid		
Lymphadenitis	8/8/1	7/7/0
Splenic necrosis	7/7/1	6/7/1
Renal, glomerular necrosis	8/6/0	6/7/1
Hepatic, focal hepatic necrosis	8/7/0	1‡/2/0
Central nervous		
Meningitis	0/4/0	2/5/1
Encephalitis	0/7/0	0/3/0

*NiV, Nipah virus. †No. animals in which the predominant lesion was observed/antigen staining was observed in that organ or tissue/vasculitis was present in that organ or tissue. For all animals, vasculitis was associated with antigen staining in blood vessel endothelium or tunica media. ‡This animal also had cholecystitis with associated viral antigen.

**Figure 5:**
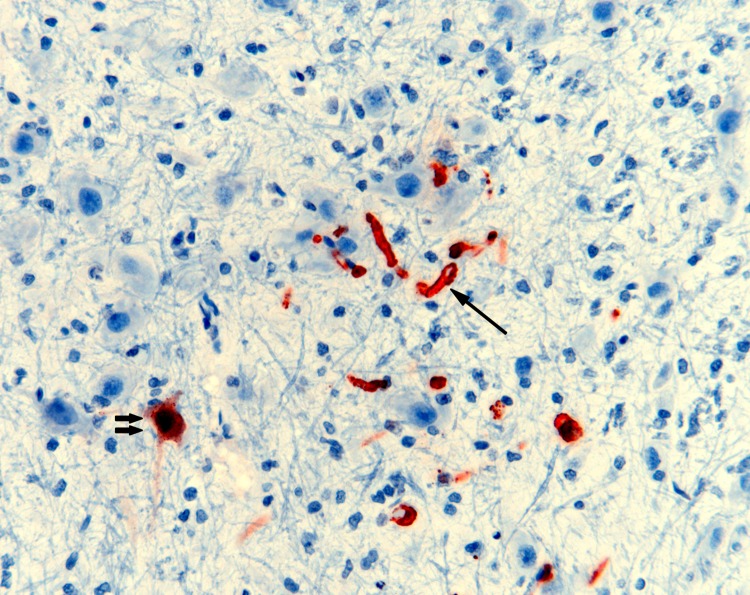
Nipah virus (NiV) antigen in neuron (double arrows) and capillary endothelia (single arrow) of a ferret experimentally infected with NiV-Bangladesh. Rabbit α-NiV N protein antiserum. Original magnification ×200.

Although encephalitis was not detected in any animal, viral antigen was found in endothelial cells within brain parenchyma of 7 and within meninges of 4 of these. Antigen was occasionally detected in neurons and glial cells adjacent to affected capillaries (Figure 5), consistent with hematogenous spread.

#### NiV-Malaysia

**Figure 6:**
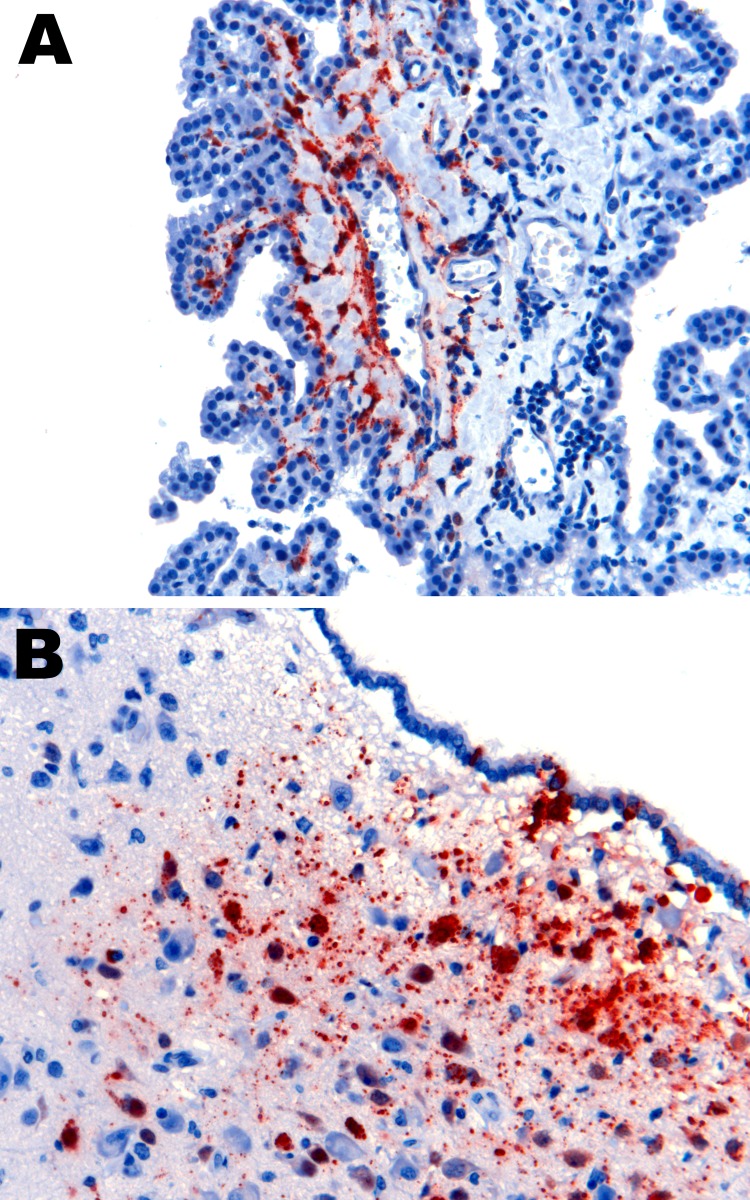
Nipah virus (NiV) antigen in ferret infected with NiV-Malaysia. A) Choroid plexus endothelium. B) ependymal epithelium and subependymal tissue, including neurons. Rabbit α-NiV N protein antiserum. Original magnification ×200.

In ferrets exposed to NiV-Malaysia, multisystemic inflammatory disease developed as described (*15*), which was generally similar to that observed in ferrets exposed to NiV-Bangladesh. Mild myocarditis was found in 1 animal and cholecystitis was found in another. Unlike findings in NiV-Bangladesh–infected ferrets, focal hepatic necrosis was found in only 1 NiV-Malaysia–infected ferret, a difference that was statistically significant (p = 0.001, Fisher exact test) but of uncertain pathogenic relevance. Mild nonsuppurative meningitis was found in 2 of 7 animals, and vasculitis in the choroid plexus was found in 1 of these. NiV antigen was identified in meningeal endothelial cells of 5 ferrets; in 3 of these 5 ferrets, it was found in the choroid plexus (Figure 6, panel A), ependyma (Figure 6, panel B), parenchymal vascular endothelium, and adjacent neurons and glia, consistent with hematogenous spread.

### Virus in Clinical Samples and Tissues at Euthanasia

Because ferret M11 did not fulfill the defined criteria for reaching humane end point attributable to NiV infection, euthanasia data for this animal were omitted from statistical analysis. Viral RNA levels in oral swab, nasal wash, and rectal swab samples and urine collected at euthanasia were comparable between ferrets infected with NiV-Bangladesh or NiV-Malaysia (by *t* test; data not shown). All animals with clinical disease had detectable viral RNA in blood at euthanasia. Mean viral load in blood at euthanasia was 10-fold higher in the NiV-Malaysia–infected group (p = 0.008; t_12_ = 3.2; difference between means: 10^1.1^ [95% CI 10^0.3^–10^1.8^]). Viral RNA was detected in virtually all tissues examined from all animals with clinical disease, and levels were generally higher in tissues from the NiV-Malaysia–infected group; this difference was significant for tissue from the olfactory pole of the brain, the nasal turbinates, pharynx, retropharyngeal lymph nodes, spleen, and bladder (Technical Appendix) and was attributed to the higher RNA levels in blood in this group at euthanasia. Although rectal shedding was detected in most animals over the course of clinical disease, virus reisolation from the jejunoileum was not successful. Virus was reisolated from rectal tissues from 1 of 6 and from 3 of 5 animals positive for viral RNA in the NiV-Bangladesh and NiV-Malaysia infection groups, respectively.

## Discussion

We compared the characteristics of viral shedding and tissue tropism between NiV-Malaysia and NiV-Bangladesh in the ferret model to examine whether these characteristics might contribute to observed differences in the clinical outcome and transmission of disease during outbreaks among humans. We found that viral shedding by nasal, oral, rectal, and urinary routes occurred in ferrets infected with both strains, as has been reported for outbreaks among humans (*23*), and we found that levels of viral genome over time were significantly higher in oral secretions from ferrets infected with NiV-Bangladesh than with NiV-Malaysia. Although this finding was not reflected by a higher rate of virus isolation in that group, an observation that was attributed at least in part to the relative insensitivity of virus isolation assay compared with RT-PCR (*24*), increasing viral RNA over time was consistent with active virus replication in the oropharynx of these animals. Accordingly, this finding in oral secretions suggests that strain differences in replication at sites relevant to transmission might occur.

Although, to our knowledge, data on viral shedding in humans over the course of infection have not been reported, epidemiologic studies identified exposure to infectious saliva or respiratory secretions from patients as a major risk factor for human-to-human transmission of NiV-Bangladesh (*13*,*25*,*26*). Lower respiratory tract involvement and associated signs, including coughing, are more commonly reported for humans infected with NiV-Bangladesh than with NiV-Malaysia and have been suggested as a contributing factor in the higher likelihood of transmission from patients so affected (*21*). We did not observe differences between the 2 strains in the form of viral antigen distribution, lesion distribution and severity, or levels of viral RNA in the oropharynx or lower respiratory tract at the time of advanced clinical disease that would offer an immediate explanation for the increased oral shedding of NiV-Bangladesh. It might be that a higher level of oral shedding of NiV-Bangladesh reflects additional, more extensive, or more efficient viral replication in the oropharynx or lower respiratory tract earlier in the infection process that is later masked by fulminant NiV infection. In addition, our criteria for euthanasia might not have reflected a consistent biological time point in the infection process with each strain. More in vivo studies of viral infection of the oropharynx and lower respiratory tract, particularly soon after exposure, are warranted to explore these points further. Differences in infection and replication efficiency between virus strains might also be elucidated by in vitro comparisons of NiV-Bangladesh and NiV-Malaysia replication kinetics in respiratory cell lines.

It is noteworthy that with both NiV strains, shedding was observed in nasal wash and oral swab samples before the onset of pyrexia, as has been reported for hamsters infected with NiV-Malaysia (*27*). This finding suggests risk for transmission during the incubation period and before hematogenous virus spread.

Isolation of virus and detection of viral RNA from rectal swab samples from animals in both infection groups supports the potential for oral–fecal NiV transmission with a comparatively higher risk for NiV-Malaysia during terminal disease. Because rectal shedding typically occurred later in the course of infection and was not associated with viral localization in the gastrointestinal tract at euthanasia, it was attributed to effusion of blood-borne virus from compromised gastrointestinal tract vasculature. The higher mean levels of viral RNA in blood at euthanasia in the group infected with NiV-Malaysia are of uncertain pathogenic significance, but it is noteworthy that increased severity of hemorrhagic diathesis was observed in this group. Thrombocytopenia and gastrointestinal bleeding have been reported for some humans with advanced NiV-Malaysia infection (*28*–*30*), and a hemorrhagic syndrome has been observed in green monkeys (*24*) and ferrets (*18*) after infection with NiV-Malaysia. In our study, animals with hemorrhagic disease tended to be those that had reached their end points for euthanasia late (9 dpi in the NiV-Bangladesh–infected group and ≥8 dpi for 4 of the 6 animals in the NiV-Malaysia–infected group), and they might have had more prolonged endothelial infection.

We were unable to assess shedding in urine over time, but virus was reisolated from urine collected at euthanasia from ferrets infected with NiV-Bangladesh and NiV-Malaysia. This finding is consistent with findings for NiV-Malaysia–infected human patients (*23*). Virus reisolation from urine and feces collected from cages containing infected animals in both groups also suggests that environmental contamination might pose an infection risk.

Viral antigen was observed in neurons and glia and in meningeal and parenchymal vascular endothelium of animals in both groups. This finding is consistent with the dual mechanism proposed for the pathogenesis of NiV neurologic disease in humans, namely, direct cellular dysfunction resulting from neuronal infection and vasculitis-associated ischemic injury to the brain (*2*,*31*–*33*).

Our observation that oropharyngeal shedding occurred at higher levels in NiV-Bangladesh–infected ferrets suggests a mechanism for the higher risk for human-to-human transmission that is observed for this NiV strain in the field, although the mechanism for enhanced shedding of NiV-Bangladesh has not yet been elucidated. However, it is recognized that heterogeneity of NiV-Bangladesh isolates (*1*,*34*) is more substantial than has been observed for NiV-Malaysia isolates. It would be of value to compare the shedding characteristics of the NiV-Bangladesh isolate studied here (from an outbreak in which human-to-human transmission was not observed) (*16*) with characteristics of isolates obtained during outbreaks with differing epidemiologic features. Transmission of NiV-Malaysia has been recently described in the hamster model (*35*), but the application of this observation to enhancing infection control with human NiV-Bangladesh is unclear, and natural transmission of NiV-Malaysia among humans occurs at low frequency (*9*,*10*). Our observations for ferrets support the view that although transmission of NiV-Malaysia between humans is possible, an increased propensity for oral shedding of NiV-Bangladesh (of pharyngeal or lower respiratory tract origin) within the context of social environmental factors in play during outbreaks of human disease leads to a higher incidence of human-to-human transmission of NiV-Bangladesh. Whether increased oral shedding of NiV-Bangladesh is predictive for increased transmissibility under controlled conditions in an animal model remains to be seen. In addition to time-course studies, in vivo studies that simulate various levels of interaction between infected and in-contact animals are warranted. On the basis of the virus shedding reported here for the ferret model, we propose that the ferret is a suitable human surrogate for further investigation of NiV transmission.

Technical Appendix Mean levels of Nipah virus RNA in tissues of respiratory tract and brain and other major organs and gastrointestinal tract of ferrets at euthanasia.
