# Supplementary material for: Transmission Routes for Nipah Virus from Malaysia and Bangladesh
Source: Emerg Infect Dis. 2012 Dec;18(12):1983–93. doi: 10.3201/eid1812.120875 (PMC3557903; doi:10.3201/eid1812.120875)
Supplement: Technical Appendix — Mean levels of Nipah virus RNA in tissues of respiratory tract and brain and other major organs and gastrointestinal tract of ferrets at euthanasia. [file 12-0875-Techapp-s1.pdf]

Article DOI: http://dx.doi.org/10.3201/eid1812.120875

Transmission Routes for Nipah Virus from Malaysia and Bangladesh

Technical Appendix

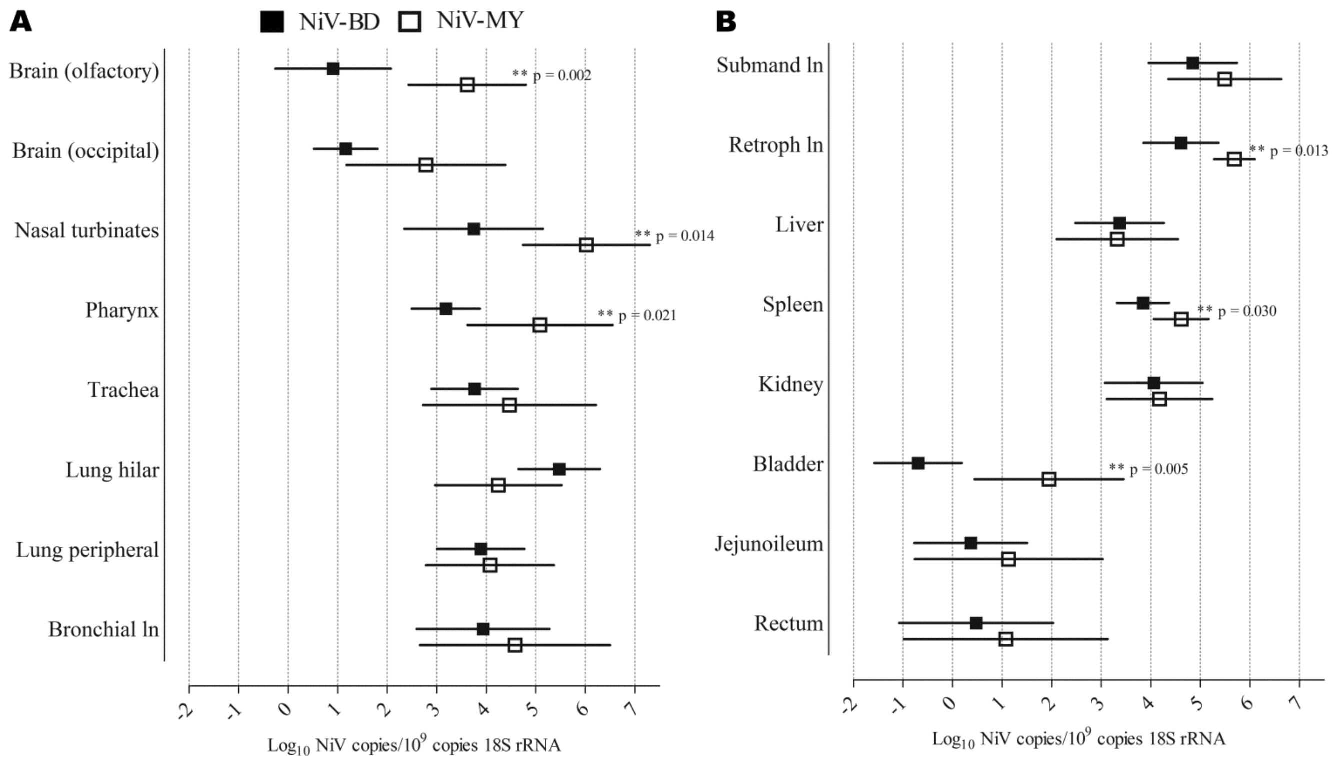

Figure. Mean Nipah virus (NiV) RNA levels in tissues of respiratory tract and brain (A) and other major organs and gastrointestinal tract (B) of ferrets at euthanasia. Differences in means were analyzed by independent samples *t* tests of transformed data, using the transformation $\log_{10}(x_2 + 0.01)$, where x_2 = NiV N gene copies per 10^9 18S rRNA copies. Vertical interval bars represent 95% CIs. BD, Bangladesh; MY, Malaysia; Submand ln, submandibular lymph nodes; Retroph ln, retropharyngeal lymph nodes.
